# Combined Tui na and Western medicine treatment improves pulmonary function and quality of life in patients with amyotrophic lateral sclerosis: A case report

**DOI:** 10.1097/MD.0000000000033612

**Published:** 2023-04-21

**Authors:** Bei Li, Haijing Liu, Cuiling Li, Meidi Yang, Tingting Zhang

**Affiliations:** a Zhijiang People’s Hospital, Yichang, China; b Yunnan University of Chinese Medicine, Kunming, China; c Zhijiang Hospital of Chinese Medicine, Yichang, China; d Hubei Provincial Hospital of Traditional Chinese Medicine, Wuhan, China; e Hubei Academy of Traditional Chinese Medicine, Wuhan, China; f Hubei University of Chinese Medicine, Wuhan, China.

**Keywords:** amyotrophic lateral sclerosis, case report, motor neuron disease, traditional Chinese medicine, Tuina

## Abstract

Amyotrophic lateral sclerosis is a rare disease that cannot be cured. We report a case of a patient with amyotrophic lateral sclerosis whose pulmonary function and quality of life were improved by a combined tui na treatment and Western medicine.

**Patient concerns:**

A 48-year-old male was diagnosed with ALS 1 year ago and was treated with western medicine and herbal medicine with no significant effect. This time, he was admitted to our department because of slurred speech, coughing and choking, and weakness of the left upper limb for more than 1 year.

**Intervention and outcome:**

After 1 month of treatment with tui na and traditional western medicine, the patient’s lung function and quality of life improved and he was discharged from the hospital.

**Diagnoses:**

Motor neuron disease. Amyotrophic lateral sclerosis.

**Lessons:**

The physiological function of ALS patients can be improved through the intervention of tui na.

## 1. Introduction

Amyotrophic lateral sclerosis (ALS) is a progressive neurodegenerative disease of unknown etiology that primarily involves motor neurons in the cerebral cortex, brainstem and spinal cord. It is also known as motor neuron disease. ALS is a rare disease for which there is no clear treatment. Once the disease is developed, death is only a matter of time.

The incidence of ALS varies from region to region. In general, the incidence rate of people in Asia is the lowest (0.73–0.94 per 100,000), followed by Europe and North America (1.71–1.89 per 100,000), while Oceania is the highest (approximately 2.25 per 100,000).^[1–3]^ This disease is characterized by muscle weakness and dysphagia, but it also has non-motor symptoms such as cognitive impairment and dysarthria. Eventually, most patients will die of respiratory paralysis or lung infection.^[4]^

Riluzole is currently the main drug used to treat ALS, but it only prolongs the life of patients by about 3 months relative to a placebo, and it does not increase muscle strength.^[5]^ The current mainstream management strategy is a multidisciplinary team approach that aims to improve the quality of life of patients and delay the deterioration of the disease as long as possible. This type of multidisciplinary team usually requires a wide range of medical practitioners, such as neurologists, nutritionists, psychologists, therapists, respiratory physicians and clinical pharmacists, to support patients and their families physically and psychologically.^[6]^

We report a case of an ALS patient that has improved pulmonary function and quality of life through a combined treatment of tuina and Western medicine.

## 2. Case presentation

A 48-year-old male patient was admitted on 29 January 2023 with slurred speech, choking cough and left upper limb weakness for more than 1 year. In November 2021, the patient developed slurred speech and a nasal voice with no apparent cause, then gradually developed weakness in the left upper limb. He was admitted to the Second Affiliated Hospital of Nanchang University in July 2022 and completed electromyography and other tests after admission. The electromyography evoked potentials indicated that the wave amplitude of the left upper limb neuromotor complex action potential was attenuated compared to the contralateral side. In August 2022, the patient went to Xuanwu Hospital of Capital Medical University for outpatient treatment and was given riluzole, certain Chinese herbs and methylcobalamin tablets as symptomatic treatment, but the symptoms of the patient did not improve significantly after taking these medicines.

Physical examination: Soft neck, mild atrophy of the left muscle of thenar, muscle strength of the left upper limb is grade 3+, right upper limb is grade 4+, proximal muscle strength of both lower limbs is grade 5-, hyperactive tendon reflexes in all 4 limbs, slightly high muscle tone in both upper limbs, double Bartholomew’s sign (−), double Hoffman’s sign (−), profound and superficial sensory and ataxic movements basically normal. On admission, the left upper arm circumference was measured at 25.0 cm, the right upper arm circumference at 25.0 cm, the left forearm circumference at 21.0 cm, the right forearm circumference at 23.0 cm, the left thigh circumference at 39.0 cm, the right thigh circumference at 40.0 cm, the left calf circumference at 36.0 cm and the right calf circumference at 36.5 cm. Pulmonary function tests: forced vital capacity: 69%, vital capacity: 65%, and maximal voluntary ventilation: 45% (Table 1). Laboratory tests showed decreased high-density lipoprotein cholesterol, increased low-density lipoprotein cholesterol, elevated Cystatin-C, decreased Complement C1q, and elevated mean platelet volume and reticulocyte percentage. The main symptoms of the patient include slurred and labored speech, residual food after meals, slowed and labored swallowing and chewing, reduced breathing ability and occasional choking and coughing when drinking water. We diagnosed him with ALS based on his medical history and clinical symptoms.

**Table 1:** Pulmonary function of the patient.

	29 Jan		8 Feb		15 Feb	
	Measured values	%Estimated value (%)	Measured values	%Estimated value (%)	Measured values	%Estimated value (%)
FVC (L)	2.60	69	2.98	80	2.87	77
FEV1 (L)	1.90	62	2.33	76	2.07	67
FEF25–75% (%)	1.57	41	2.10	56	1.50	40
PEF (L/s)	3.06	38	4.90	61	4.62	58
MEF25% (L/s)	0.7	42	0.91	55	0.55	33
MEF50% (L/s)	1.78	41	2.47	58	1.94	45
MEF75% (L/s)	2.95	42	4.68	67	3.86	55
VC (L)	2.53	65	2.94	75	3.18	82
IC (L)	2.73	3	1.14	42	1.90	70
MVV (L/min)	52.4	45	66.8	58	63.1	55

Pulmonary function tests on 29 January, 8 and 15 February.

BEV = VCVital capacity, FEF25–75% = forced expiratory flow from 25 to 75% of FVC, FEV1 = forced expiratory volume in one second, FVC = forced vital capacity, IC = inspiratory capacity, MEF = maximal expiratory flow, MVV = maximal voluntary ventilation, PEF = peak expiratory flow.

After admission, we treated the patient with a combination of Chinese and Western medicine. This includes oral riluzole tablets (50 mg per 12 h) and Tuina (a form of Chinese manual therapy) treatment. We treated the patient daily with Tuina therapy. Specific methods are shown in Table 2.

**Table 2:** Specific Tui Na method.

Tui na area	Patient-doctor posture	Tui na order	Tui Na manipulation	Pressing acupressure points
Head and face	The patient is in the supine position with the doctor sitting on one side	Forehead to cheeks Top of head to tip of ears Both sides of the head Face	Extrapolation method, rubbing method	Baihui (GV20), Fengchi (GB20)
Upper limb	The patient is lying on his side and the doctor is standing on the affected side	Around the shoulder joint, then in turn to the posterior, lateral and anterior sides of the upper limb Shoulder, elbow and wrist joints	Rolling method, handle method	Jianyu (LI15), Binao (LI14), Quchi (LI11), Shou sanli (LI10)
Low back and posterior aspect of lower limbs	The patient is in prone position with the doctor standing on the affected side	The spinal area is from top to bottom Lumbosacral and posterior aspect of the lower limbs	Extrapolation method, rolling method	Huatuojiaji, Eight-liao, Huantiao (GB30), Chengfu (BL36), Yinmen (UB37), Weizhong (BL40), Chengshan (BL57)
Anterior and lateral lower limbs	The patient lies on his back with the doctor standing on the affected side	Lateral, anterolateral and medial to the affected limb Hip, knee and ankle joints	Rolling method, handle method	Biguan (ST31), Fengshi (GB31), futu (ST32), Liangqiu (ST34), DUbi (ST35), Zusanli (ST36), Jiexi (ST41), Sanyinjiao (SP6), Xuehai (SP10), Neixiyan (EX-LE4)

**Figure 1.**
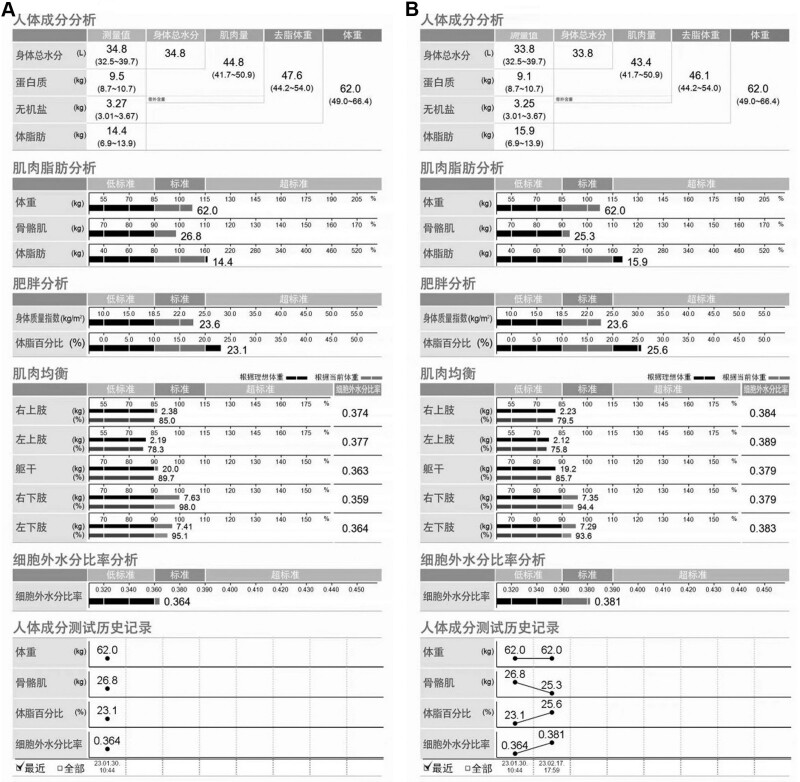
Body composition test on 30 Jan (A) and 17 Feb (B).

On the second day of admission, we performed a body composition test which showed 26.8 kg of skeletal muscle, 14.4 kg of body fat and a body mass percentage of 23.1% (Fig. 1A). On 8 February, 2023, a second pulmonary function test was carried out and this time the patient’s forced vital capacity, vital capacity and maximal voluntary ventilation were all improved (Table 1). We tested the patient’s pulmonary function for the third time on 15 February, 2023 (Table 1). The results show a decrease from the second time, but still higher than the first test. On 17 February, 2023, a second body composition test was performed on the patient (Fig. 1B). The patient’s skeletal muscle was reduced to 25.3 kg but body fat was elevated to 15.9 kg and body mass percentage increased to 25.6%, with varying degrees of muscle loss in the limbs and trunk.

**Figure 2.**
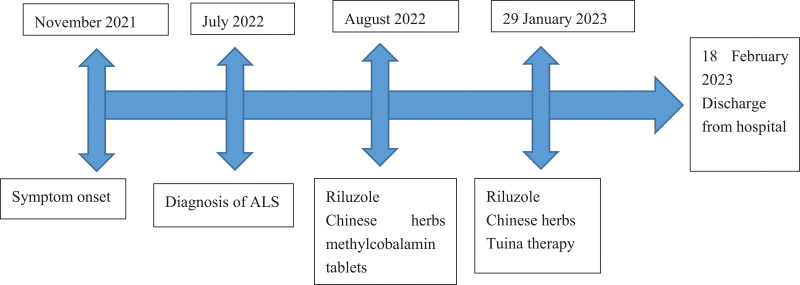
Timeline.

On 18 February, 2023, we examined the patient’s left upper arm circumference of 25.9 cm, right upper arm circumference of 26.1 cm, left forearm circumference of 21.0 cm, right forearm circumference of 22.5 cm, left thigh circumference of 39.1 cm, right thigh circumference of 40.5 cm, left calf circumference of 37.6 cm and right calf circumference of 37.4 cm. Compared to the measurements taken on admission, all limbs, except the left forearm, showed varying degrees of increase in circumference. The patient described that his slurred speech, choking on drinking water and left upper limb weakness were slightly improved, and tongue stirring speed and strength were improved after the treatment. The patient decided to be discharged on the same day. The timeline for this patient is shown in Figure 2.

The reporting of this study conforms to the CARE guidelines and was approved by the Clinical Research Ethics Committee of Zhijiang People’s Hospital. Written informed consent was obtained from the patient to publish this case report and accompanying images.

## 3. Discussion

As a rare disease, ALS has 3 major challenges: it is difficult to diagnose, it is difficult to cure, and it has a low survival rate. To date, there is no gold standard for diagnosing ALS, and the diagnosis of ALS is mainly based on a combination of clinical symptoms, blood tests, imaging, nerve conduction tests, electromyography and other ancillary tests, many of which are used to rule out other diseases rather than to diagnose ALS.^[7,8]^ So far, there is still no effective approach to treat ALS. Riluzole, while considered functional somehow, merely extends the life of patients by about 3 months after the diagnosis, and it does not make any improvement in the quality of life of patients.^[5]^ Thus, while some medical practitioners are working on more effective drugs, there is also a portion of the medical profession trying to slow down the deterioration of patients and improve their quality of life by various means. Clinical guidelines for ALS have also been issued in many countries to support multidisciplinary combination therapy to improve the quality of life of patients.^[9]^

As the disease progresses, patients with ALS develop many other problems, such as muscle spasms, pain and depression.^[10]^ This reinforces the need for us to support patients through a multidisciplinary team to minimize their suffering. In addition to western medicine treatment, Chinese physicians also exerted the expertise of traditional medicine to provide new ideas for curing ALS patients.

In China, ALS patients often receive a combination of Chinese and Western medical treatments. Which includes Western medicines, Chinese herbs (decoctions or compounds), acupuncture, Tuina, and so on.^[11]^ Chinese herbal medicine has been used in China for thousands of years, and most Chinese people believe that Chinese herbal medicine is effective for many diseases, there are a very limited number of practitioners outside China that use Chinese herbal medicine. Therefore, there is very little literature on this subject from other countries.^[12]^

Tuina treatment is a green therapy and is more comfortable for many patients, so it is more accessible to patients. Exercise is thought to enhance function and slow down the course of the disease in patients with ALS.^[13]^ Tuina (or Chinese massage) is also a passive exercise that can work on multiple symptoms in the management of ALS.^[14]^ Kim sungha’s research found that the most frequent use of mind and body medicine in South Korean patients with ALS was Tuina treatment.^[15]^ The use of Tuina treatment varies from region to region due to differences in health insurance policies and medical culture.^[16]^ Compared to other countries, China has the highest proportion of ALS patients receiving Tuina treatment.^[11]^

When we treat patients with Tuina, we not only massage the muscles but also apply pressure to the acupuncture points (Table 2). Electroacupuncture is thought to have anti-inflammatory effects in the treatment of patients with ALS.^[17,18]^ Pressing the acupuncture points with the fingers is a stimulation of the points as well as the electroacupuncture. In our case, we treated the patient using Western medicine and Tuina for 30 days. The patient’s pulmonary function was partially improved, limb circumference was increased, and some improvements in clinical discomfort were achieved. This provides some insight into how we can improve the quality of life of people with ALS.

However, it should be noted that the patient’s pulmonary function was unstable, rising and then falling. Although the results from the third test show improvements when compared to those from the first test, it is very difficult to predicate how long the effects of this treatment will be maintained. It is well known that ALS patients have progressive exacerbations and therefore the duration of maintenance of this treatment is crucial to the progression of the disease. Therefore, in this case, we need to follow the patient’s quality of life on an ongoing basis in order to assess the long-term outcome. In the meantime, the patient should be kept on treatment as long as possible to achieve the full benefits of this combined treatment.

Due to the wide range of symptoms of ALS, symptomatic support is currently the more appropriate management method. Our literature search also revealed that at this stage, it may be in the patient’s best interest to use different approaches for different symptoms (Table 3).

**Table 3:** Summary of treatment for ALS.

References	Gender	Age	Main symptoms	Treatment	Duration of treatment	Outcome
Portaro et al^[19]^	Female	69	Upper limb muscles weakness	Physiotherapy plus robotics	2 mo	Temporary improvement
Clemente^[20]^	Male	64	Lower limb spasms, dyspnea, dysphagia	Ultramicronized Palmitoylethanolamide	45 d	Temporary improvement
Białkowska et al^[21]^	Male	71	Tetraparesis with resultant minor distal weakness	Neurofeedback after Stem cell transplantation	30 d	Temporary improvement
Sharma et al^[22]^	Male	40	Muscle atrophy of the limbs and weakness of the neck muscles	Cell therapy and neurorehabilitation	16 mo	Temporary improvement
Hassan et al^[23]^	Male	52	Unable to grip and falls frequently	Stem cell therapy	Twice (2 d)	Loss of motor function after 6 mo
Urata et al^[24]^	Male	75	Dropped head syndrome	Short-term intensive exercise program	2 wk	Temporary improvement
Lu gaochen et al^[25]^	Female	48	Weakness of the limbs	Washed microbiota transplantation	1 yr	Maintain the status quo

ALS = amyotrophic lateral sclerosis.

## 4. Conclusion

As ALS requires lifelong treatment and there are no effective drugs available, once diagnosed, patients should seek every likely approach in the treatment of the disease. Until effective drugs are identified, the intervention of traditional Chinese medicine offers a new treatment option for patients, thereby providing ALS patients with certain relief both physically and psychologically. It should be noted that the sample size in this report is very small, so it can only provide the medical community with some clues rather than strong evidence for the treatment of ALS. We look forward to more basic and clinical studies to support our findings for the management of ALS.

## Author contributions

**Conceptualization:** Bei Li, Tingting Zhang.

**Data curation:** Meidi Yang.

**Formal analysis:** Bei Li, Haijing Liu.

**Investigation:** Bei Li, Tingting Zhang.

**Methodology:** Bei Li, Tingting Zhang.

**Writing – original draft:** Bei Li, Cuiling Li.

**Writing – review & editing:** Haijing Liu.
